# Spatiotemporal precision interventions for cardiac repair and regenerative therapy

**DOI:** 10.1038/s12276-026-01704-4

**Published:** 2026-05-01

**Authors:** Mei Hua Ting, Huipeng Zhang, Siyuan Liu, Peng Wang, Hongjun Li, Wenbin Zhang, Junbo Ge, Ning Zhang

**Affiliations:** 1 Department of Cardiology, Sir Run Run Shaw Hospital, Zhejiang University School of Medicine, Hangzhou, China; 2 Zhejiang Key Laboratory of Cardiovascular Intervention and Precision Medicine, Hangzhou, China; 3 Engineering Research Center for Cardiovascular Innovative Devices of Zhejiang Province, Hangzhou, China; 4 State Key Laboratory of Advanced Drug Delivery and Release Systems, Key Laboratory of Advanced Drug Delivery Systems of Zhejiang Province, Liangzhu Laboratory, School of Pharmacy, Zhejiang University, Hangzhou, China; 5 Department of Hepatobiliary and Pancreatic Surgery, The Second Affiliated Hospital, School of Medicine, Zhejiang University, Hangzhou, China; 6 Department of Cardiology, Zhongshan Hospital, Fudan University, Shanghai, China; 7 Shanghai Institute of Cardiovascular Diseases, Shanghai, China; 8 State Key Laboratory of Cardiovascular Diseases, Shanghai, China; 9 National Clinical Research Center for Intervention Medicine, Shanghai, China

**Keywords:** Translational research, Drug delivery

## Abstract

Restoring cardiac function after myocardial infarction remains a major challenge, as current pharmacological and interventional therapies primarily mitigate symptoms and slow disease progression without addressing the irreversible loss of functional myocardium. Although a diverse range of biologically active agents has been developed to modulate inflammation, angiogenesis, fibrosis, and cardiomyocyte survival, their therapeutic impact is frequently limited by delivery strategies that fail to match the dynamic and heterogeneous nature of post-infarction healing. Advances in biomaterials, nanotechnology, and device engineering have enabled drug delivery systems capable of spatiotemporally programmed therapeutic engagement. By responding to injury-associated cues, recreating key features of the myocardial microenvironment, and incorporating programmable release architectures, these systems coordinate localization, release kinetics, and duration of action with distinct phases and regions of cardiac repair. When combined with appropriate delivery interfaces, including nanocarriers, injectable depots, structured platforms, and biologically derived vehicles, spatiotemporal drug delivery transforms therapy from passive administration into an active determinant of biological outcome. This Review synthesizes recent mechanistic and engineering advances to frame spatiotemporal precision as a unifying principle for cardiac drug delivery. Aligning therapeutic action with the intrinsic biology of myocardial healing provides a rational pathway toward more effective, durable, and biologically informed strategies for cardiac repair and, where biology permits, regeneration.

## Spatiotemporal strategies transform cardiac therapy approaches

Ischemic heart disease results in the permanent loss of cardiomyocytes after myocardial infarction, leading to heart failure. Current treatments focus on symptom relief rather than tissue restoration, hindered by ineffective delivery to the injured myocardium. This Review explores drug delivery strategies for cardiac repair, emphasizing the importance of aligning therapeutic presence with the dynamic biological demands of post-infarction healing. The authors discuss smart biomaterials that interact with injury-associated microenvironments, enabling context-dependent therapeutic control. They highlight significant findings, such as the use of stimuli-responsive biomaterials that leverage injury-associated signals to regulate therapeutic activation. These advancements are crucial as they offer a framework for more precise and personalized cardiac therapy. Future directions include integrating biosensor systems for real-time release and developing scalable, cell-free vesicle-based therapies, potentially accelerating clinical translation and improving patient outcomes.

This summary was initially drafted using artificial intelligence, then revised and fact-checked by the author.

## Introduction

Ischemic heart disease remains the leading cause of mortality worldwide, accounting for more than nine million deaths each year^1^. Despite substantial advances in pharmacological therapy and revascularization procedures that have substantially improved survival after myocardial infarction (MI), these interventions mainly alleviate symptoms and slow disease progression rather than restoring lost myocardial tissue. The permanent loss of cardiomyocytes following infarction triggers maladaptive remodeling processes that progressively impair ventricular function and ultimately lead to heart failure (HF). This challenge is further complicated by the limited regenerative capacity of the adult heart, in which endogenous repair responses are modest, transient, and quickly replaced by fibrotic scar formation^2^.

Efforts to transition from symptomatic treatment toward myocardial repair have therefore driven the development of various therapeutic approaches aimed at modulating inflammation, promoting angiogenesis, limiting fibrosis, and preserving cardiac function^3^. These strategies include various therapeutic cargoes such as small molecules, proteins, nucleic acids, and cell-based or vesicle-based agents. Despite encouraging biological rationale and preclinical efficacy, many of these therapies share a common limitation: ineffective delivery to the injured myocardium. Rapid systemic clearance, off-target biodistribution, and poor localization to infarcted tissue continue to constrain therapeutic impact, making drug delivery a central bottleneck in cardiac repair and regeneration.

**Fig. 1:**
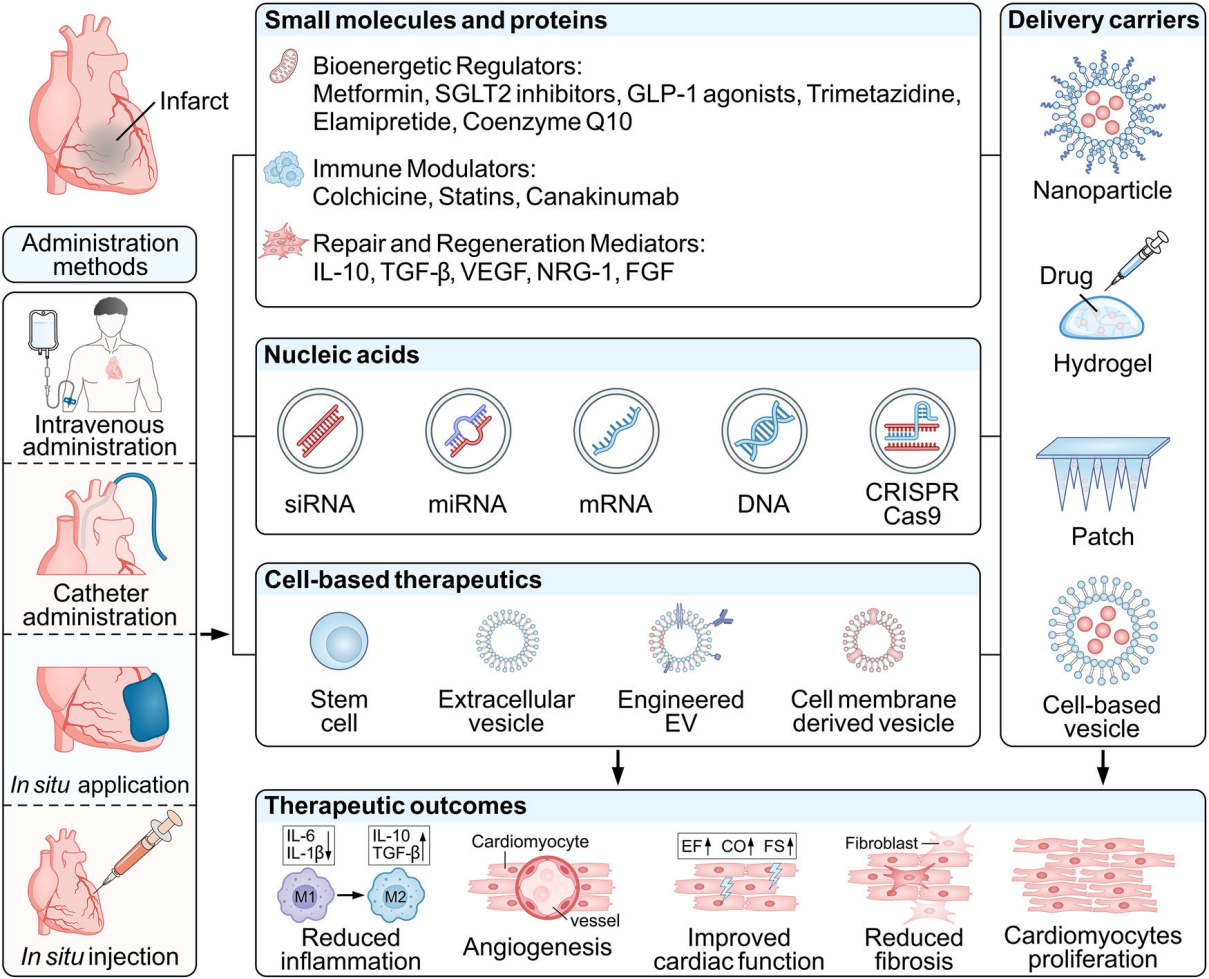
Integrative therapeutic landscape for spatiotemporal cardiac repair enabled by smart biomaterials. This schematic summarizes the solution space in which diverse therapeutic cargoes, including small molecules, nucleic acids, and cell-derived or vesicle-derived agents, are integrated with advanced delivery platforms such as nanoparticles, injectable hydrogels, patches, and bioinspired carriers to achieve spatially and temporally aligned intervention after myocardial infarction. Delivery interfaces span systemic, catheter-based, and local in situ routes, enabling differential engagement with key reparative processes including inflammation modulation, angiogenesis, and fibrotic remodeling across distinct post-infarction phases. Rather than presupposing specific mechanisms or outcomes, the figure illustrates how biomaterial-enabled delivery platforms provide a unifying framework to match therapeutic action with the evolving biological demands of the injured myocardium. EV extracellular vesicle, miRNA microRNA, siRNA small interfering RNA, TGF-β transforming growth factor, VEGF vascular endothelial growth factor, NRG-1 neuregulin-1, FGF fibroblast growth factor, CO cardiac output, FS fractional shortening.

Alongside this change in therapeutic goals, drug delivery systems (DDS) have become a key focus, leading to various approaches aimed at enhancing myocardial localization and retention. These strategies encompass different delivery modalities, administration routes, and therapeutic objectives (Fig. 1). Nanoparticles, injectable hydrogels, patches, microneedle arrays, and biologically driven carriers have all been explored as vehicles to improve cardiac targeting, whereas systemic, catheter-based, and local delivery routes offer differing balances between accessibility and spatial control^4,5^. These approaches have expanded the therapeutic toolkit, bridging the gap between biological potential and effective engagement of injured myocardium.

However, successful cardiac repair depends not only on what is delivered but on when, where, and under what biological conditions therapy is active. Post-infarction healing is governed by tightly coupled temporal dynamics and pronounced spatial heterogeneity^6^, which impose constraints that static or uniform delivery strategies are poorly equipped to meet. Aligning therapeutic presence with these evolving biological demands has therefore emerged as a defining challenge for next-generation cardiac DDS.

In this Review, we examine drug delivery strategies for cardiac repair through the lens of spatiotemporal biological alignment. We first define the temporal and spatial constraints imposed by post-infarction healing, highlighting why conventional delivery paradigms often fail to match the dynamic requirements of injured myocardium. We then discuss how smart biomaterials can interact with injury-associated microenvironments to enable context-dependent therapeutic control, followed by an overview of delivery interfaces that support precise deployment within the heart. Finally, we address translational considerations and future directions necessary to advance spatiotemporally informed DDS toward clinical application.

## Biological constraints of the post-infarction therapeutic window

**Fig. 2:**
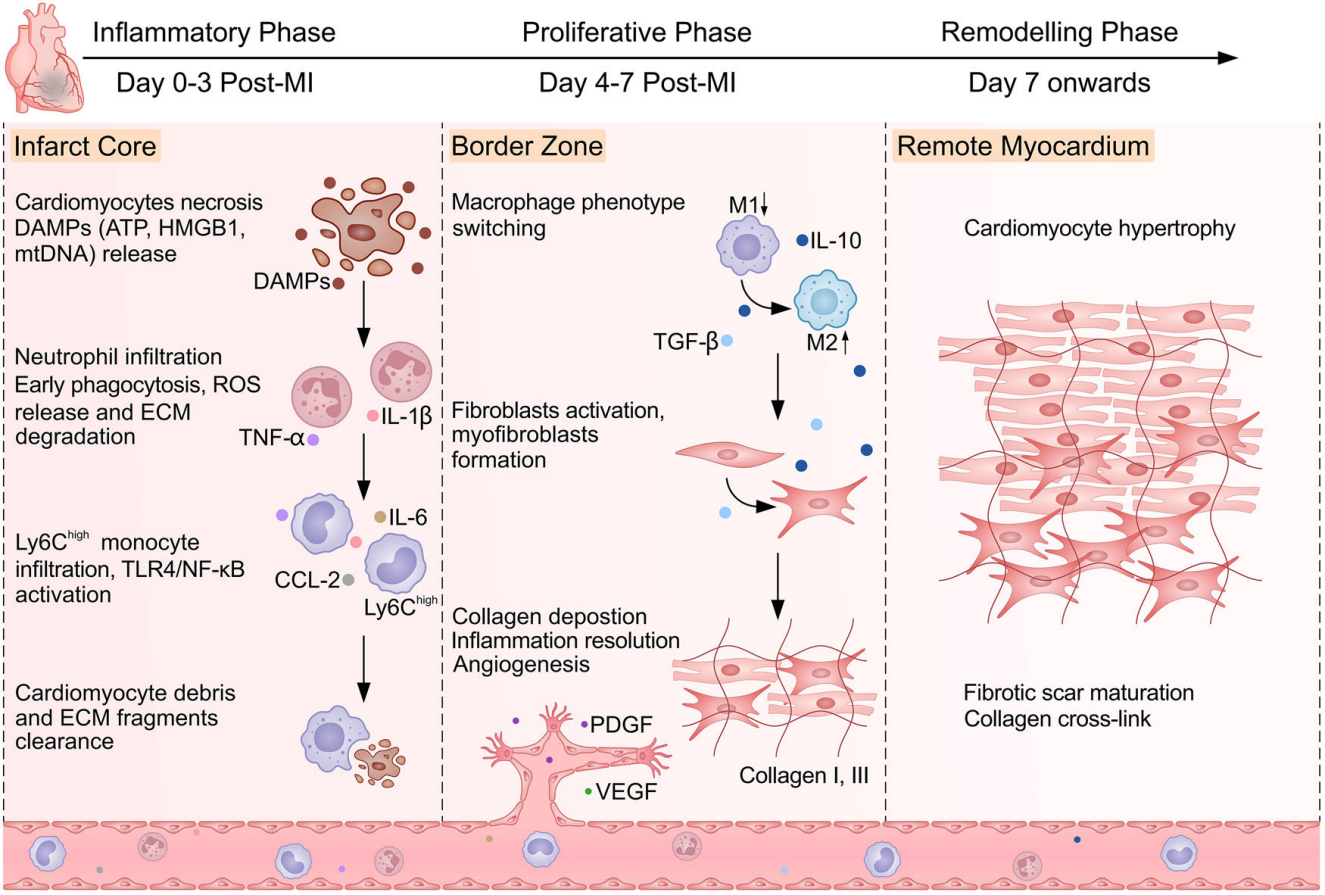
Spatiotemporal progression of myocardial infarction and post-infarction healing. The myocardial response to infarction unfolds through overlapping inflammatory (days 0–3), proliferative (days 4–7), and remodeling (day 7 onwards) phases, each characterized by distinct immune, cellular, and extracellular matrix dynamics. In the infarct core, cardiomyocyte necrosis triggers damage-associated molecular pattern (DAMP) signaling, acute inflammatory cell infiltration, oxidative stress, and matrix degradation, followed by fibroblast activation and scar formation. The peri-infarct border zone represents a biologically active interface in which immune modulation, angiogenesis, and matrix remodeling coexist, supporting tissue stabilization but remaining sensitive to dysregulated intervention. By contrast, the remote myocardium undergoes progressive secondary remodeling driven by altered loading and neurohumoral signaling, including cardiomyocyte hypertrophy and interstitial fibrosis, which impair contractile and electrical function. These temporally and spatially heterogeneous processes define a dynamic therapeutic landscape that constrains the timing, localization, and duration of effective intervention. ECM extracellular matrix, MI myocardial infarction, mtDNA mitochondrial DNA, NF-κB nuclear factor-κB, PDGF platelet-derived growth factor, ROS reactive oxygen species, TGF-β transforming growth factor-beta, TLR Toll-like receptor, TNF-α tumor necrosis factor-alpha, VEGF vascular endothelial growth factor.

MI triggers overlapping temporal phases and spatially heterogeneous responses that define a dynamic therapeutic window. As a result, therapeutic success depends not just on the choice of agent but also on whether its activity coincides with the evolving biological demands of the injured heart. Defining this therapeutic window is therefore essential for ensuring that intervention remains effective, safe, and durable (Fig. 2).

### Temporal constraints of myocardial healing

The temporal progression of myocardial repair following infarction is often described as overlapping inflammatory, proliferative, and remodeling phases rather than a strictly sequential cascade^7,8^. During the early inflammatory period, neutrophils and pro-inflammatory macrophages infiltrate and clear necrotic tissue, a response that is essential for initiating repair, but if excessive or prolonged, exacerbates cardiomyocyte loss and adverse remodeling^6^. Therapeutic intervention in this window thus requires precise temporal alignment, as premature suppression of inflammation may hinder debris clearance, whereas delayed modulation may fail to prevent secondary injury. As inflammation subsides, the myocardium enters a proliferative phase characterized by fibroblast activation, endothelial expansion, and extracellular matrix (ECM) deposition that together support granulation tissue formation, neovascularization, and preservation of ventricular geometry^7,9^. Concurrently, a collagen-rich matrix is assembled and organized, laying the structural foundation for subsequent scar maturation. The remodeling phase is dominated by maturation of the collagenous scar and ventricular restructuring^10^. Fibrosis is indispensable for mechanical stability and prevention of rupture, yet its dysregulation compromises compliance, disrupts electrical conduction, and promotes HF and arrhythmia. Therapeutic strategies must therefore support reparative fibrosis during proliferation while constraining maladaptive expansion during remodeling, a requirement that is poorly met by fixed or uniform drug kinetics.

Beyond reparative processes, the potential for myocardial regeneration in the adult heart is also tightly time-limited. Unlike the neonatal myocardium, where robust regenerative responses can occur^11^, adult cardiomyocytes display only a brief and modest capacity for cell cycle re-entry after infarction before reverting to a terminally differentiated state. Early post-infarction stages may briefly permit regenerative-like responses, including cardiomyocyte plasticity and permissive immune signaling. However, these features wane as inflammation resolves, ECM accumulates, and scar maturation advances^12^. As fibrotic remodeling consolidates, the myocardial environment becomes increasingly refractory to regenerative intervention, implying that therapies in supporting cardiac regeneration must be delivered within a narrow temporal window^13^.

### Spatial constraints of myocardial healing

Myocardial healing after infarction is profoundly shaped by spatial heterogeneity across the injured heart. The infarcted myocardium is not a uniform biological entity but comprises discrete regions that differ in cellular composition, perfusion, mechanical properties, and signaling environment^8,14^, such that interventions that are beneficial in one compartment may be ineffective in another.

The infarct core is characterized by extensive cardiomyocyte necrosis, severe hypoxia, oxidative stress, and loss of structural integrity^2^. Early inflammatory infiltration and matrix degradation are followed by fibroblast activation and collagen deposition, whereas limited perfusion and elevated interstitial pressure restrict molecular transport and dampen therapeutic accessibility^15^. As healing progresses, the core stabilizes into a dense, collagen-rich scar that secures mechanical stability at the expense of cellular plasticity, rendering it largely refractory to late-stage biological intervention.

Surrounding the core, the peri-infarct or border zone forms a transitional microenvironment with partial tissue viability and heightened biological activity. Cardiomyocytes here are structurally compromised but salvageable, and dynamic interactions among endothelial cells, immune cells, and activated fibroblasts regulate angiogenesis, matrix remodeling, and inflammatory resolution. This region, therefore, constitutes a critical spatial niche for modulating functional recovery^14,16^ but is also sensitive to inappropriate exposure, as excessive intervention can exacerbate remodeling or disrupt adaptive repair. Beyond the injured regions, the remote myocardium initially preserves structural integrity but undergoes secondary remodeling driven by altered loading and neurohormonal signaling. Compensatory hypertrophy, interstitial fibrosis, and conduction changes progressively contribute to ventricular dysfunction^17^, raising the risk of unintended consequences when therapies distribute broadly beyond their intended target.

### Spatiotemporal interdependence

Conceptually, endogenous repair and regeneration require developmental-like spatiotemporal coordination. During cardiac development, tissue growth depends on synchronized cellular competence, patterning, and mechanical maturation^18^. By contrast, post-MI healing unfolds in a disrupted landscape of short-lived plasticity windows and fibrotic stabilization. Regenerative failure stems not from absent molecular programmes but from their fundamental spatiotemporal misalignment^18^. This synchronization between time and space creates a moving therapeutic target that static, uniformly distributed therapies cannot adequately address. Effective modulation of post-infarct healing therefore requires strategies that synchronize therapeutic action with both temporal progression and regional context, aligning intervention with the appropriate biological state in the appropriate myocardial compartment.

## Smart biomaterial frameworks for spatiotemporal control

The biological constraints outlined in the section “Biological constraints of the post-infarction therapeutic window” reveal a fundamental limitation of conventional delivery approaches. Smart biomaterials have emerged as a central strategy for revolutionizing cardiac healing therapies. These materials are engineered to sense injury-associated cues, interact with surrounding tissue, and regulate therapeutic availability in a context-dependent manner. By coupling material behavior to endogenous biochemical or cellular signals, smart biomaterials enable therapeutic activity to be synchronized with both the temporal progression of healing and the spatial organization of the injured myocardium.

### Stimuli-responsive biomaterials

The infarcted myocardium is characterized by dynamic and spatially heterogeneous biochemical cues, such as oxidative stress, local acidosis, and elevated enzymatic activity^19^. These injury-associated signals provide endogenous triggers that can be harnessed to regulate material behavior and therapeutic activation. Stimuli-responsive biomaterials exploit this principle by combining pathological microenvironment changes to controlled material transformation or cargo release.

Reactive oxygen species (ROS) is among the earliest and most spatially confined signal following MI owing to ischemia/reperfusion injury, inflammatory cell infiltration, and mitochondrial dysfunction. ROS-responsive biomaterials leverage this redox imbalance through oxidation-sensitive chemistries or intrinsic antioxidant functionality, enabling focal activation within acutely injured tissue^20–22^. Such systems match the early phase, during which redox normalization can preserve cardiomyocyte viability and stabilize the reparative niche. However, the short temporal window could limit the durability of ROS-triggered activation.

Local acidosis is a broader and more persistent feature of the injured myocardium, extending across both the infarct core and border regions during inflammation and early proliferation. pH-responsive biomaterials typically exploit protonation-dependent transitions or acid-labile linkages to achieve regional and sustained therapeutic engagement^23,24^. Compared with ROS-responsive platforms, these systems exhibit reduced focal specificity but offer prolonged responsiveness across a larger injury territory. Importantly, many pH-responsive materials also modulate the acidic microenvironment itself, positioning them as regulators of tissue context rather than simple release switches^25^. As remodeling progresses and fibrosis advances, pH gradients would gradually diminish.

Matrix metalloproteinases are a class of enzymes that control ECM turnover during post-infarction healing. Matrix metalloproteinase-responsive biomaterials incorporate protease-cleavable motifs that enable activation specifically within regions of active remodeling^26^. These systems coupled therapeutic delivery to matrix dynamics, offering phase-selective access to proliferative and remodeling niches, where intervention must balance structural stabilization against maladaptive fibrosis. As scar maturation progresses, enzymatic activity decreases, as does the functionality of these systems, defining a later but finite therapeutic window.

### Biomimetic and biointeractive materials

An emerging class of smart biomaterials is designed to actively engage the cardiac microenvironment and shape the repair processes. Biomimetic and biointeractive materials aim to recreate key biochemical, mechanical, and biophysical features of native myocardium while dynamically interfacing with resident and infiltrating cells, extending DDS from passive transport vehicles to tissue-level behavioral regulation.

The most intuitive biomimetic approach is to recapitulate elements of the native ECM, which provides both structural support and instructive signaling during cardiac development and repair. Decellularized ECM (dECM)-derived hydrogels preserve tissue-specific matrix proteins, proteoglycans, and glycosaminoglycans, enabling modulation of immune responses, promotion of angiogenesis, and support of endothelial and cardiomyocyte survival^27^. Cardiac dECM retains biochemical cues that can influence cell-fate decisions and, in some contexts, partially re-engages developmental signaling pathways associated with cardiomyocyte plasticity^28^. These properties position dECM-based systems as biologically rich scaffolds that extend beyond mechanical reinforcement to provide instructive microenvironments^29^.

Synthetic ECM mimetics offer complementary advantages through precise control over material composition and properties. Platforms based on gelatin methacryloyl, polyethylene glycol (PEG), hyaluronic acid and derivatives, and related polymers can be engineered with tunable stiffness, degradation kinetics, and ligand presentation to match the evolving mechanical landscape of the infarcted heart^30,31^. They can influence cardiomyocyte mechanosensing, fibroblast activation, immune cell behavior, and endothelial organization, thereby indirectly shaping remodeling trajectories^32^. Their modularity further enables integration of therapeutic cargoes while maintaining reproducible and well-defined architectures.

Many biointeractive materials are also designed to actively modulate the inflammatory microenvironment. The phenotype and persistence of infiltrating immune cells influence the balance between reparative and maladaptive remodeling. Biomaterials functionalized with immunomodulatory peptides, cytokine-binding domains, or small-molecule signals have been shown to promote macrophage polarization toward reparative states, facilitating inflammation resolution and limiting excessive fibrosis^33,34^. Electrical and mechanical uncoupling are hallmark features of fibrotic myocardium that contribute to arrhythmogenesis and contractile dysfunction. To address this, electroconductive biomaterials incorporating conductive polymers or nanomaterials have been developed to restore local signal propagation and mechanical synchrony^35^. These materials influence cell alignment, maturation, and survival by acting as mechanoelectrical interfaces.

### Programmable and sequential delivery systems

Although stimuli-responsive and biointeractive biomaterials enable context-aware activation and microenvironment regulation, many regenerative strategies require coordinated intervention across multiple stages of healing. Programmable delivery systems encode temporal control directly into material architecture, allowing therapeutic release to proceed in a predefined or condition-dependent sequence without external intervention. In MI, sequential systems are advantageous as early inflammatory modulation, intermediate support of angiogenesis and tissue stabilization, and later attenuation of fibrotic remodeling, enabling phase-specific engagement that more closely mirrors endogenous repair dynamics.

A common strategy involves hierarchical material organization, in which multiple cargoes are embedded within compartments exhibiting distinct degradation or diffusion kinetics^36^. For example, fast-releasing components can provide early antioxidant or anti-inflammatory therapies, whereas slower-degrading matrices sustain pro-angiogenic or cytoprotective cues during proliferation^37–39^. More advanced designs integrate stimuli-responsive elements, allowing environmental cues such as ROS, pH, or enzymatic activity to gate individual release steps^40,41^. Sequential systems also benefit from integration with biomimetic and biointeractive materials. They can simultaneously control when therapy is delivered and how the tissue responds by embedding programmable release within matrices that modulate immune responses, mechanosensing, or electromechanical coupling.

Programmable DDS represent a convergence toward intelligent and personalized therapeutic platforms that synchronize material behaviors, biological context, and therapeutic intent. These approaches move cardiac DDS beyond single-event intervention toward coordinated modulation of myocardial healing (Table 1).

**Table 1:** Smart biomaterial strategies for spatiotemporal regulation of cardiac repair.

Biomaterial category	Material platform	Injury-associated cue	Therapeutic function/mechanism	Biological context	Primary spatiotemporal role
**Stimuli-responsive biomaterials**	Reactive oxygen species (ROS)-responsive	ROS; ischemia/reperfusion injury; acute inflammation	Redox-sensitive degradation or intrinsic antioxidant activity enabling targeted cytoprotection	Acute phase; infarct core and early reperfused regions	Early-phase gating: aligns intervention with transient oxidative stress to preserve cardiomyocyte viability
	pH-responsive	Local acidosis due to hypoxia and altered metabolism	Protonation-dependent destabilization or acid-labile release of sustained therapeutic cues	Inflammatory to early proliferative phases; infarct core and border zone	Sustained regional engagement: maintains responsiveness across extended injury territories
	Matrix metalloproteinase (MMP)-responsive	MMP activity during extracellular matrix (ECM) remodeling	Protease-cleavable motifs enabling phase-selective activation and matrix-coupled delivery	Proliferative to remodeling phases; regions of active ECM turnover	Phase-selective access: targets remodeling niches while sparing mature scar tissue
**Biomimetic and biointeractive materials**	Decellularized ECM (dECM)	Native biochemical cues (ECM proteins, proteoglycans, GAGs)	Preservation of tissue-specific signaling that supports immune modulation and cellular plasticity	Peri-infarct border zone; reparative niche	Instructive scaffold: recapitulates developmental-like signaling environments
	Synthetic ECM mimetics (for example, gelatin methacryloyl, polyethylene glycol, hyaluronic acid)	Altered tissue mechanics and wall stress	Tunable stiffness, degradation, and ligand presentation to modulate mechanotransduction	Evolving mechanical landscape during remodeling	Modular support: indirectly shapes remodeling trajectories by regulating mechanosensing
	Conductive/biointeractive materials	Electromechanical uncoupling in fibrotic myocardium	Restoration of electrical conductivity and mechanoelectrical synchrony	Fibrotic and arrhythmogenic regions	Active regulation: integrates electrical and mechanical repair with local delivery
**Programmable and sequential delivery**	Hierarchical release systems	Overlapping biological phase transitions	Multicargo, staged release matched to inflammatory, proliferative, and remodeling phases	Whole infarct life cycle	Coordinated phase control: enables temporally ordered therapeutic engagement
	Integrated multimodal platforms	Combined biochemical and mechanical cues	Coupling stimuli-responsiveness with biomimetic matrices for context-aware release	Dynamic post-infarction microenvironment	Synchronized modulation: moves beyond single-event intervention toward system-level control

This table summarizes major classes of smart biomaterials used to achieve spatially and temporally controlled therapeutic engagement after myocardial infarction. Strategies are organized according to their dominant injury-associated cues, material platforms, and mechanisms of action, alongside the biological contexts in which they operate and their primary spatiotemporal roles during post-infarction healing. These approaches illustrate how endogenous signals, matrix interactions, and programmable release architectures can be leveraged to align therapeutic activity with phase-specific and region-specific demands of myocardial repair. *GAG* glycosaminoglycans.

## Delivery interfaces for spatiotemporal cardiac therapy

Smart biomaterials permit spatiotemporally programmed therapeutic behavior but their effectiveness ultimately depends on how they are delivered to and retained within the injured heart. Delivery modalities therefore serve as the critical interface between material design and biological outcome, shaping myocardial exposure, localization, residence time, and safety^42^. A range of delivery formats, including systemic nanocarriers, injectable depots, and structured platforms, have been developed to translate material intelligence into effective cardiac engagement. Increasing integration with catheter-based, image-guided, and minimally invasive techniques has further expanded the translational scope of these approaches^4,43^. This section examines the principal delivery interfaces and emphasis is placed on how physical format, route of administration, and targeting strategy jointly determine effective myocardial engagement within dynamic post-infarction environment.

### Nanoparticles

Nanoparticles are one of the most versatile and widely explored delivery interfaces for cardiac repair, offering scalable manufacture, modular design, and compatibility with minimally invasive administration^44,45^. Their nanoscale dimensions enable vascular transport, tissue penetration, and intracellular delivery, positioning them as a primary vehicle for the programmed deployment of biomaterials within the injured heart.

Polymeric nanoparticles, most commonly based on poly(lactic-co-glycolic acid), PEG derivatives, and related biodegradable systems, have been extensively investigated for cardiac applications. These platforms enable controlled release, cargo protection, and tunable degradation kinetics, supporting delivery of a wide variety of drugs, such as small molecules, proteins, and nucleic acids^46,47^. Lipid-based nanoparticles, including liposomes and lipid nanoparticles (LNP), offer advantageous intracellular delivery, especially for nucleic acid therapeutics. Accelerated by clinical translation of mRNA vaccines^48^, LNP technologies have been adapted for cardiac delivery of mRNA, small interfering RNA, microRNA, and CRISPR-based genome-editing systems aimed at modulating cardiomyocyte survival, angiogenesis, immune activation, and fibroblast behavior^45,49^. Their clinical translation and modular lipid composition make LNP especially attractive for translational cardiac applications.

Targeting strategies within nanoparticle delivery encompass both active and passive mechanisms. Active targeting relies on surface-conjugated ligands such as antibodies, peptides, or small molecules that bind injury-associated markers (for example, fibroblast activation protein, periostin, selectins, or angiogenic integrins)^50–53^, enhancing localization to infarcted or inflamed regions. Passive targeting exploits altered vascular permeability, immune cell recruitment, and inflammatory trafficking within the injured myocardium, allowing nanoparticles to preferentially accumulate without explicit targeting motifs^45,54^.

Emerging approaches increasingly demonstrate that cardiac retention can be programmed through nanocarrier composition, complementing traditional ligand-based and physical targeting strategies. Selective organ targeting (SORT) LNPs exemplified this paradigm, wherein modulation of helper lipid identity and charge biases protein corona formation, endothelial interactions, and immune responses, thereby reshaping biodistribution following systemic administration^49,55^. Although SORT strategies have been extensively validated for other organs, such as the liver, lungs, and spleen, their underlying principles suggest mechanistic plausibility for myocardial tropism via formulation enhancements, representing an underexplored yet promising direction for cardiac nanomedicine.

Despite these advances, nanoparticle delivery to the heart remains constrained by systemic clearance, hepatic uptake, renal filtration, and rapid myocardial washout^5^. Design refinements, including PEGylation^56^, stimuli-responsive surface chemistries, and biomimetic cloaking with platelet-derived or stem-cell-derived membranes have been used to enhance circulation time, immune evasion, and cardiac tropism^57,58^. Increasingly, nanoparticles are also deployed as components within hybrid systems, such as injectable hydrogels, microneedle arrays, or patch-based depots, in which local retention and spatiotemporal control can further be amplified.

### Cardiac patches and microneedle platforms

Although nanoparticle-based systems excel in systemic delivery and intracellular access, they remain constrained by myocardial washout, limited tissue residence, and dependence on vascular permeability. Cardiac patches and microneedle platforms address these limitations by operating at the tissue scale, establishing direct physical contact with the injured tissue to achieve durable, spatially confined therapeutic exposure. Rather than competing with nanoparticles, they occupy a complementary position within the delivery landscape.

Cardiac patches anchor therapeutic function to the epicardial surface, transforming delivery from a transient circulatory event into a sustained local interaction. These patches provide a stable platform for integrating mechanical reinforcement, localized release, and electrical interfacing to the injured ventricle^59^. Contemporary designs extend far beyond passive scaffolding, incorporating dECM, synthetic polymers, conductive elements, and embedded nanocarriers or microcarriers to create multifunctional therapeutic surfaces that actively shape the repair environment^60^.

The historical reliance on open-chest implantation has often framed cardiac patches as surgically burdensome. However, recent engineering advances have shifted this perception. Flexible, conformable patches with catheter-based or pericardial deployment, along with sprayable and self-assembling systems, have expanded the feasibility of epicardial delivery without compromising spatial precision. Microneedle platforms further refine tissue-level interfacing by introducing controlled penetration across the epicardial or endocardial surface. Arrays of biodegradable microneedles create transient microchannels that bypass diffusion barriers and deliver therapeutic cargo directly into myocardial tissue at defined depths^61^. This architecture enables precise spatial dosing, enhances retention, and reduces off-target exposure, while remaining compatible with minimally invasive access routes^62^. Importantly, microneedles also provide a structural framework for incorporating sequential release, stimuli responsiveness, or electrically active components, linking physical access with temporal control^63^.

### Injectable hydrogels

Injectable hydrogels occupy a unique middle ground within the cardiac delivery paradigm, combining the procedural accessibility of minimally invasive injection with the local retention and structural influence of tissue-scale platforms. They do not rely on vascular transport or permanent implantation but are deployed as flowable precursors that undergo in situ gelation, conforming to the complex geometry of the infarcted myocardium and establishing a localized therapeutic niche^35,64^. From a delivery perspective, injectable hydrogels offer a pragmatic balance between precision and feasibility. They can be administered via percutaneous, catheter-guided, or direct myocardial injection, allowing localization while remaining compatible with established interventional cardiology workflows.

These hydrogels can adapt to the dynamic mechanical and biological environment of the injured heart. Shear-thinning behavior enables catheter-based or transendocardial injection, whereas rapid gelation, triggered by temperature, enzymatic activity, ionic interactions, or chemical crosslinking, anchors the material within myocardial tissue despite continuous contractile motion^65,66^. This capacity to transition from injectable fluid to stable depot allows hydrogels to achieve sustained myocardial accumulation without the need for open surgical access^67^.

Besides serving as physical depots, these materials, by tuning stiffness, degradation kinetics, and porosity, influence cell infiltration, fibroblast activation, and immune responses, thereby modulating the post-MI microenvironment^68,69^. Many systems are designed to soften or degrade in synchrony with tissue healing, ensuring that mechanical support and therapeutic presence evolve alongside endogenous repair processes rather than imposing static constraints^70^. Similar to patches and microneedle arrays, injectable hydrogels are amenable to multifunctional integration. Therapeutic cargoes can be embedded within the matrix or within secondary carriers dispersed throughout the gel, enabling sequential and stimuli-responsive release profiles, as well as co-delivery of synergistic signals that address inflammation, angiogenesis, fibrosis, and electrical instability in a coordinated manner^32,71,72^. Incorporation of conductive polymers, immunomodulatory motifs, or bioadhesive chemistries further expands the functional repertoire of these platforms.

### Engineered cell-based and vesicle-based delivery systems

Bioinspired vesicle-derived and membrane-derived DDS represent a rapidly advancing class of interfaces that exploit endogenous biological trafficking mechanisms to enhance myocardial targeting and retention. These strategies leverage the intrinsic capacity of living cells or cell-derived vesicles to confer immune compatibility, injury homing, and intercellular delivery, independent of cellular engraftment or tissue replacement.

Extracellular vesicles (EVs), including exosomes and microvesicles, have emerged as key mediators of paracrine signaling in cardiac repair^73,74^. Secreted by a wide range of cell types, EVs encapsulate protein, lipids, and nucleic acids that modulate inflammation, angiogenesis, and cardiomyocyte survival. In preclinical models of MI, EVs derived from mesenchymal stromal cells^25,75^, cardiac progenitor cells^76^, and induced pluripotent stem cell-derived^77^ sources have demonstrated cardioprotective and reparative effects, primarily through their immunomodulatory and pro-angiogenic cargo. Their nanoscale size, intrinsic biocompatibility, and low immunogenicity further position EVs as versatile carriers for cardiac therapy (Table 2).

**Table 2:** Characteristics of extracellular vesicles and exosome-mimetic nanovesicles.

Characteristics	Exosomes	EMNVs	Microvesicles	Apoptotic bodies
**Size**	30–150 nm	50–200 nm	100–1000 nm	1000–5000 nm
**Origin**	Multivesicular bodies	Cell-derived membrane components (engineered)	Plasma membrane	Plasma membrane
**Biogenesis/fabrication**	Inward budding of endosomal membranes and released by exocytosis	Top-down fabrication via extrusion, sonication, freeze–thaw cycles, or microfluidics	Budding or shedding directly from plasma membrane	Fragmentation of apoptotic cells into vesicles
**Cargo profiles**	Proteins, lipids, nucleic acids	Drugs, synthetic cargo, proteins, nucleic acids	Proteins, lipids, nucleic acids	Cell organelles, proteins, lipids, nucleic acids
**Cellular uptake mechanism**	Endocytosis, phagocytosis	Endocytosis, membrane fusion	Endocytosis, membrane fusion	Phagocytosis
**Immunogenicity**	Low	Low^a^	Moderate^a^	Moderate to high
**Biodegradability**	High	High	High	High

Comparison of major extracellular vesicle subtypes, including exosomes, microvesicles, and apoptotic bodies, and engineered exosome-mimetic nanovesicles (EMNVs), highlighting differences in biogenesis, size range, cargo composition, cellular uptake mechanisms, immunogenicity, and translational considerations relevant to spatiotemporally controlled cardiac drug delivery.

^a^Depends on membrane source, purification, and surface modification.

To enhance therapeutic consistency and targeting specificity, efforts have focused on engineering EV cargo and surface properties. Donor cell preconditioning, such as hypoxia, inflammatory priming, or metabolic stress, can enrich vesicles with reparative microRNAs or stress-adaptive proteins^78–80^. Genetic modification of parent cells further enabled controlled loading of defined cargoes^81,82^, whereas surface functionalization with cardiac-homing peptides or injury-responsive ligands improves myocardial targeting following systemic or regional administration^83^. These approaches increasingly position EVs as programmable delivery vehicles rather than a passive by-product of cell therapy.

Recognizing the challenges of EV heterogeneity, the scalable manufacturing of membrane-derived and vesicle-mimetic systems has made these translationally tractable alternatives. Exosome-mimetic nanovesicles, generated through top-down extrusion or sonication methods, recapitulate key structural and functional features of EVs while enabling standardized production and cargo loading^84^. In parallel, membrane-coated nanoparticles, using platelet, immune cells, or stem cell membranes, transfer endogenous injury-homing and immune-evasive properties onto a synthetic core, achieving biological targeting without introducing living cells^57,75,85,86^. With their size, flexibility, and biocompatibility, they are often integrated with other delivery interfaces mentioned in the previous section, functioning as mobile, biologically adaptive reservoirs that cooperate with endogenous repair processes while maintaining design flexibility.

## Clinical and translational considerations

Despite promising efficacy in preclinical models, the clinical translation of spatiotemporal controlled DDS for cardiac healing remains limited. These barriers arise not only from the complexity of cardiovascular pathology but also from regulatory, manufacturing, and delivery challenges intrinsic to sophisticated biomaterial systems.

**Fig. 3:**
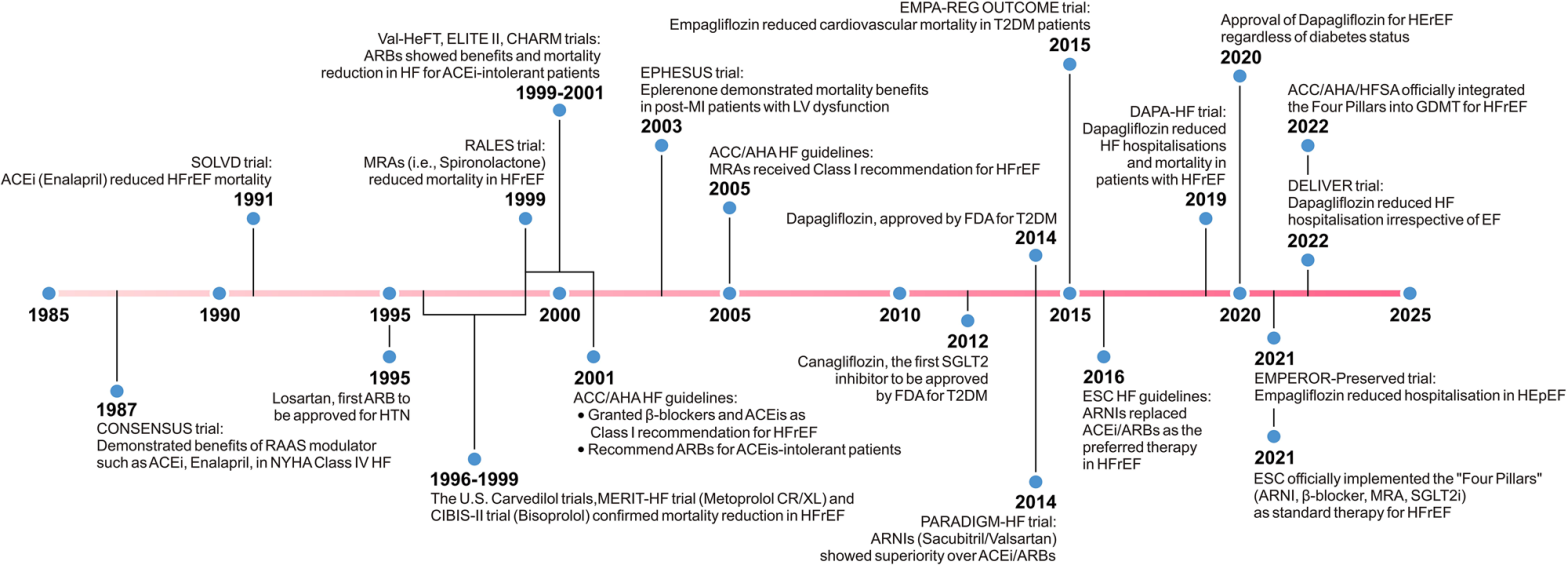
Evolution of pharmacological management in heart failure with reduced ejection fraction. This timeline highlights key clinical trials, guideline endorsements, and regulatory approvals that have shaped contemporary HFrEF management. Several drug classes now central to GDMT, including RAAS modulators, β-blockers, MRAs, and SGLT2i, were initially developed for other indications and later repurposed or expanded following mechanistic and clinical insights. The progressive incorporation of ARNIs and SGLT2i reflects a shift toward cardiometabolic and multisystem modulation rather than disease-specific targeting alone. Collectively, this evolution illustrates how therapeutic impact can be transformed without introducing new molecular entities, providing a precedent for reformulating existing cardiovascular drugs using advanced DDS to improve myocardial selectivity, reduce off-target exposure, and further personalize therapy. ACC/AHA American College of Cardiology/American Heart Association, ACEI angiotensin-converting enzyme inhibitor, ARB angiotensin receptor blocker, ARNI angiotensin receptor–neprilysin inhibitor, DDS drug delivery system, EF ejection fraction, ESC European Society for Cardiology, GDMT guideline-directed medical therapy, HFpEF heart failure with preserved ejection fraction, HFrEF heart failure with reduced ejection fraction, HTN hypertension, LV left ventricle, MI myocardial infarction, MRA mineralocorticoid receptor antagonist, NYHA New York Heart Association, RAAS renin–angiotensin–aldosterone system, SGLT2i sodium-glucose cotransporter 2 inhibitor, T2DM type 2 diabetes mellitus.

A translational reference point is the evolution of pharmacological therapy for heart failure with reduced ejection fraction, where successive waves of guideline-directed medical therapy have progressively improved outcomes through systemic administration^87,88^ (Fig. 3). Although these agents have revolutionized HF management, their efficacy is often limited by dose-limiting side effects, off-target exposure, and suboptimal myocardial selectivity. Spatiotemporally programmed DDS offer a complementary strategy to enhance the therapeutic index of established drugs by improving localization, prolonging myocardial residence, and reducing systemic burden.

In this context, reformulation of FDA-approved cardiovascular agents with advanced DDS platforms is a pragmatic translational pathway. Rather than introducing new molecular entities, this approach leverages well-characterized safety profiles to accelerate clinical adoption^89,90^. Several clinically approved or widely used drugs, such as SGLT2 inhibitors^91,92^, metformin^93^, colchicine^94^, and statins^95^, exhibit pleiotropic cardioprotective, immunomodulatory, or anti-fibrotic effects beyond their original indications. Encapsulation within nanoparticles, hydrogels, or locally retained depots could enable spatially confined and temporally appropriate delivery to injured myocardium, mitigating systemic toxicity while preserving therapeutic efficacy. Such strategies align closely with regulatory expectations and may shorten development timelines compared with first-in-class biomaterials or gene therapies.

Apart from repurposing existing drugs, advances in molecular and cellular biology are expanding the spectrum of therapeutic targets relevant to post-infarction repair. Increasing evidence highlights metabolic reprogramming, mitochondrial health, and epigenetic regulation as key determinants of cardiomyocyte survival, plasticity, and maladaptive remodeling following MI. Shifts in substrate utilization, particularly fatty acid oxidation, influence cellular stress tolerance and energetic efficiency in the injured heart^96,97^. Similarly, regulation of mitochondrial dynamics and autophagy has emerged as a critical mechanism for preserving cardiomyocyte viability and metabolic flexibility^85,98^. At the epigenetic level, chromatin remodeling and transcriptional reprogramming are being actively explored to suppress pathological gene expression or to enable direct cardiac reprogramming for advanced fibrosis refractory to regenerative interventions^99–101^.

Translational feasibility also depends on delivery logistics and manufacturability. Systemic administration offers procedural simplicity but suffers from nonspecific biodistribution and hepatic clearance, whereas local delivery approaches achieve superior myocardial retention at the cost of invasiveness. Catheter-based and image-guided techniques provide an important middle ground, enabling regional precision within established interventional workflows. From a manufacturing perspective, platform complexity remains a key concern. Systems involving living cells, multicomponent hybrids, or poorly defined biological materials face challenges in batch-to-batch consistency, sterilization, and regulatory characterization. Modular and cell-free platforms, such as synthetic nanoparticles, injectable hydrogels, and engineered vesicle mimetics, offer more tractable pathways toward scalable production and regulatory approval.

## Future directions and outlook

**Fig. 4:**
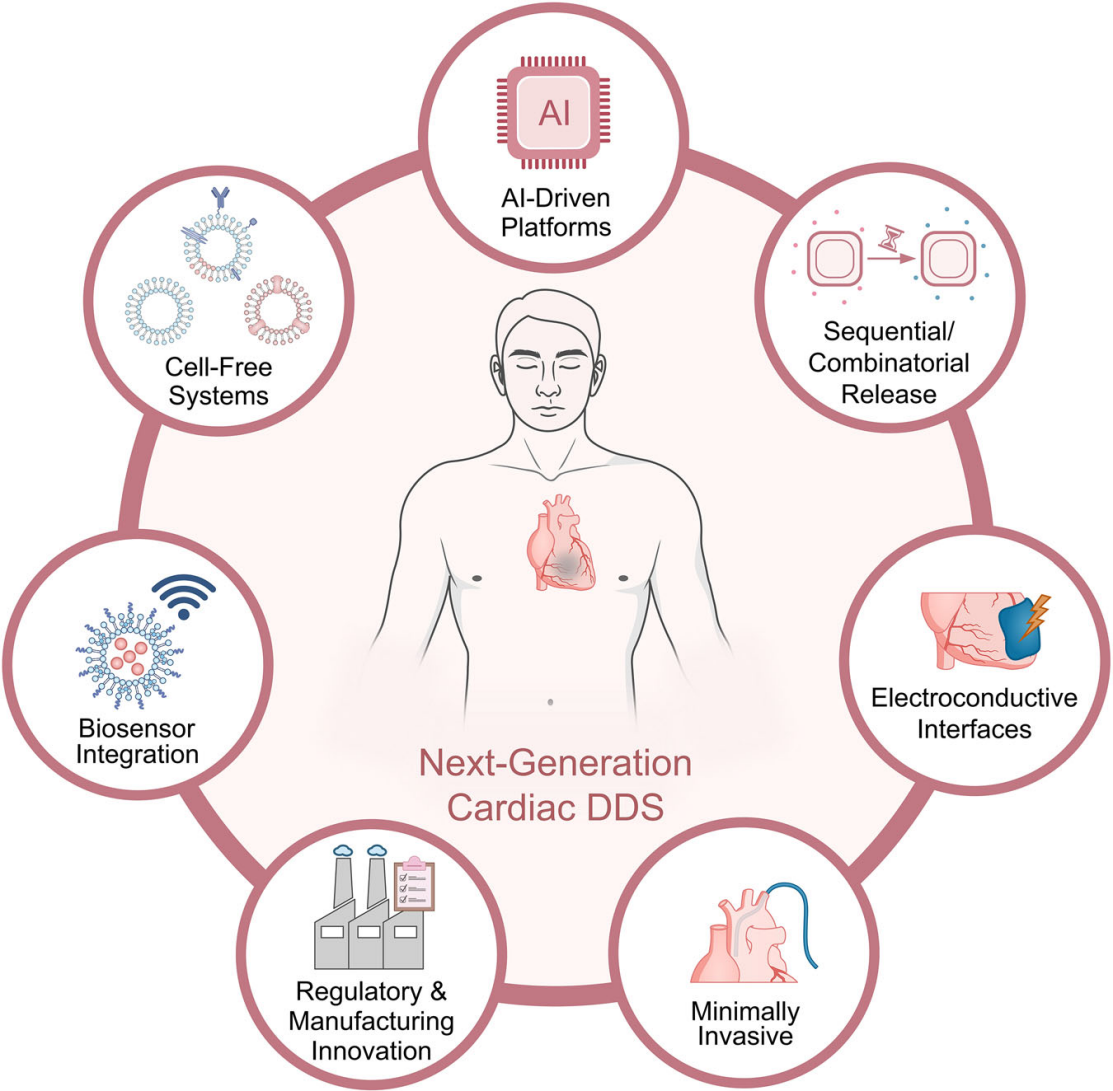
Future directions in spatiotemporal drug delivery for cardiac repair and regeneration. Next-generation spatiotemporal drug delivery systems (DDS) are evolving toward multifunctional and adaptive platforms capable of aligning therapeutic activity with the dynamic biology of post-infarction healing. Key trajectories include biosensor-integrated systems that enable real-time, feedback-controlled release; phase-specific combinatorial delivery platforms synchronized with inflammatory, proliferative, and remodeling stages; electroconductive biomaterials that support electromechanical coordination; and scalable, cell-free vesicle-based therapies that enhance translational feasibility. Parallel advances in minimally invasive delivery technologies, artificial intelligence (AI)-guided design, and harmonized regulatory and manufacturing frameworks are expected to accelerate clinical translation and support more precise, personalized cardiac therapy.

Spatiotemporally controlled DDS are entering a phase of conceptual maturation, evolving from responsive carriers into integrated platforms capable of coordinating therapeutic action across the complex and dynamic landscape of myocardial repair. Rather than addressing individual pathological features in isolation, next-generation DDS are increasingly designed to operate as programmable systems that integrate sensing, timing, localization, and biological feedback. This shift reflects a broader convergence of biomaterials science, systems biology, and interventional cardiology, redefining how therapeutic precision may be achieved in cardiac repair and regeneration (Fig. 4).

One major trajectory is the integration of temporal orchestration into DDS design. Future platforms are expected to move beyond single-phase responsiveness toward staged or hierarchically programmed release profiles that align with successive phases of myocardial healing^102^. By synchronizing immunomodulatory, pro-angiogenic, and antifibrotic cues with evolving biological states, such systems aim to minimize therapeutic interference while maximizing cumulative benefit. Importantly, this temporal programming is increasingly encoded at the material level through degradation kinetics, compartmentalized architectures, or multiresponsive chemistries, reducing reliance on external intervention^103^.

In parallel, bioelectrical and biomechanical integration is emerging as a defining feature of advanced cardiac DDS. Conductive biomaterials and electroactive scaffolds are being developed not only to deliver therapeutics but also to restore electromechanical continuity across injured myocardium. These interfaces enable coordinated electrical signaling, promote cardiomyocyte alignment and maturation, and offer opportunities for stimulus-responsive release. As understanding of mechanoelectrical coupling in cardiac repair deepens, such platforms may serve as multifunctional hubs that couple structural support, signal propagation, and therapeutic modulation within a single construct^59^.

Another key direction is the continued transition toward cell-free yet biologically intelligent systems. Engineered EVs, membrane-coated nanoparticles, and exosome-mimetic constructs exemplify efforts to capture the instructive capacity of living cells while avoiding the variability, scalability, and safety concerns associated with cellular therapies. These platforms preserve endogenous targeting mechanisms and paracrine signaling while offering greater control over composition, reproducibility, and regulatory characterization. Their modularity further enables integration with hydrogels, microneedle arrays, or injectable depots, expanding their applicability across acute and chronic cardiac settings.

Looking ahead, minimally invasive compatibility will be a decisive determinant of clinical relevance. DDS that can be deployed via catheter-based, percutaneous, or image-guided approaches are better positioned for integration into contemporary interventional workflows. Paintable scaffolds, injectable matrices, and microneedle-based interfaces exemplify efforts to reconcile spatial precision with procedural feasibility^62,71^. As these delivery formats mature, their combination with advanced imaging and mapping technologies may further enhance localization and therapeutic control without increasing procedural burden.

Finally, the increasing complexity of spatiotemporal DDS underscores the need for system-level design frameworks. Artificial intelligence, predictive modeling, and data-driven optimization are poised to support material selection, release profile tuning, and patient-specific adaptation^104,105^. When integrated with high-resolution imaging, omics data sets, and longitudinal biomarkers, these tools may enable rational design of DDS tailored to individual healing trajectories rather than population averages. Importantly, such approaches will require parallel advances in standardization, manufacturing, and regulatory harmonization to ensure that technological sophistication translates into clinical impact.

## Conclusion

The development of DDS for cardiac repair is increasingly shaped by the understanding that therapeutic efficacy depends not only on the choice of agent but also on the timing, location, and biological context in which it is active within the injured heart. Post-infarction healing is neither spatially uniform nor temporally linear; rather, it unfolds across overlapping biological phases and heterogeneous myocardial regions, creating a dynamic therapeutic landscape that static delivery paradigms are ill-suited to address. This Review therefore positions spatiotemporal control as a central strategy for aligning therapeutic intervention with the evolving biology of myocardial repair.

Recent advances illustrate how such alignment can be realized in practice. Stimuli-responsive biomaterials, biointeractive matrices, and programmable release systems harness endogenous cues and phase-specific dynamics to regulate therapeutic localization, duration, and intensity. When combined with appropriate delivery interfaces, these platforms transform DDS from passive carriers into active modulators of repair, capable of coordinating inflammatory resolution, angiogenesis, and remodeling processes. In this context, spatiotemporal precision emerges not as a discrete technology but as a unifying design principle that amplifies the impact of both established pharmacotherapies and emerging molecular repair strategies.

Crucially, spatiotemporally programmed DDS also offer a pragmatic route toward clinical impact by extending the utility of existing cardiovascular drugs. Enhanced myocardial localization and reduced systemic exposure may enable the repurposing or re-deployment of approved agents in patients who cannot tolerate conventional systemic dosing because of off-target effects or comorbidities. Moreover, the capacity to tune release kinetics and spatial engagement provides a framework for accommodating patient-to-patient variability in injury severity, healing trajectories, and microenvironmental responses. In this way, spatiotemporal DDS support a shift toward more precise and personalized cardiac therapy, refining how therapies are deployed rather than replacing them outright.
